# Diversity and Antifungal Susceptibility of *Malassezia* spp. Isolated From Brazilian Patients With Pityriasis Versicolor and Seborrheic Dermatitis

**DOI:** 10.1111/myc.70171

**Published:** 2026-03-27

**Authors:** Diogo Coelho de Pádua Oliveira, Ana Paula Possa, Ana Raquel de Oliveira Santos, Ana Kleiber P. Borges, Patrícia Silva Cisalpino, Raquel Vilela, Carlos Augusto Rosa, Susana Johann

**Affiliations:** ^1^ Departamento de Microbiologia, Instituto de Ciências Biológicas Universidade Federal de Minas Gerais Belo Horizonte MG Brazil; ^2^ Campus Palmas Universidade Federal de Tocantins TO Brazil; ^3^ Faculdade de Farmácia Universidade Federal de Minas Gerais Belo Horizonte MG Brazil

**Keywords:** antifungal susceptibility, *M. japonica*, *M. yamatoensis*, *Malassezia* species, *Pityriasis versicolor*, *Seborrheic dermatitis*

## Abstract

**Background:**

*Malassezia* spp. are part of the microbiota of many animals, including humans. However, under certain conditions, they can become pathogenic. Diseases associated with *Malassezia* include pityriasis versicolor (PV), seborrheic dermatitis (SD), Malassezia folliculitis, atopic dermatitis, psoriasis and fungemia.

**Objective:**

The present study aimed to describe the distribution of *Malassezia* species among Brazilian patients with PV and SD and to evaluate their susceptibility profiles to common antifungals.

**Methods:**

In this study, 102 clinical samples from patients with PV or SD were analysed. Clinical isolates of *Malassezia* were identified at the species level by sequencing the D1/D2 variable domains of the large subunit rRNA gene. Antifungal susceptibility was assessed using a modified microbroth dilution method adapted for the growth of *Malassezia* species.

**Results:**

Among the 40 cultures obtained, six *Malassezia* species were identified. *M. furfur* was the most prevalent species (40.0%), followed by *M. sympodialis* (27.5%), *M. globosa* (15.0%), *M. japonica* (7.5%) and both *M. yamatoensis* and *M. slooffiae* (5.0% each). All isolates exhibited high MICs to caspofungin (> s16 μg/mL) and to isoconazole (MIC₅₀ = 8 μg/mL). Miconazole and clotrimazole showed MIC₅₀ values of 4 μg/mL, while itraconazole and ketoconazole were more active, with an MIC₅₀ of 0.125 μg/mL.

**Conclusion:**

This study showed the diversity of *Malassezia* species causing PV and SD in Brazil, including the rare species *M. yamatoensis* and *M. japonica* . These findings highlight the importance of antifungal susceptibility testing for these species to guide appropriate therapy.

## 1 Introduction

The genus *Malassezia* comprises lipophilic yeasts (such as *M. pachydermatis*), and most species are lipid‐dependent [1]. These yeasts are part of the microbiota of many animals, including humans, where they live as commensal organisms on the skin and scalp. However, under certain conditions, they may become pathogenic due to predisposing factors related to both the cutaneous microenvironment and alterations in the host immune system [2, 3, 4].

Diseases associated with *Malassezia* spp. include pityriasis versicolor (PV), seborrheic dermatitis (SD), *Malassezia* folliculitis, atopic dermatitis, psoriasis and fungemia, particularly in premature neonates receiving parenteral nutrition [5, 6, 7, 8, 9]. More recent studies have also shown that this genus may be involved in the pathogenesis of certain types of cancer, such as pancreatic ductal adenocarcinoma and breast tumours [10, 11, 12]. Pancreatic ductal adenocarcinoma (PDA) tumours in both humans and murine models exhibit an approximately 3000‐fold increase in fungal burden compared with normal pancreatic tissue, with a pronounced enrichment of *Malassezia* spp. In this context, activation of the complement cascade via ligation of mannose‐binding lectin (MBL), which recognises glycans on the fungal cell wall, has been shown to be essential for oncogenic progression [10]. Beyond pancreatic cancer, *M. globosa* is highly abundant in breast cancer and is capable of colonising mammary tissue, contributing to increased tumour incidence and reduced survival in mice, likely through activation of the IL‐17A/macrophage axis and induction of SPHK1‐mediated proliferation of breast cancer cells [12]. In parallel, *M. restricta* has been implicated in inflammatory bowel disease, as it is enriched in patients with Crohn's disease and induces CARD9‐dependent innate inflammatory responses and exacerbation of colitis in mouse models [13].

Treatment of *Malassezia*‐associated skin diseases is usually carried out with topical agents, such as ketoconazole shampoo or miconazole cream. For widespread or treatment‐resistant lesions, systemic therapy with fluconazole or itraconazole may be used. In inflammatory skin conditions associated with *Malassezia* infection, treatment is often supplemented with anti‐inflammatory therapy [14, 15, 16].

To date, 19 species of *Malassezia* have been described. Among them, *M. globosa* , *M. restricta*, *M. obtusa* , *M. slooffiae*, *M. sympodialis*, *M. furfur*, *M. dermatis*, *M. japonica* , *M. yamatoensis*, *M. arunalokei* and *M. polysorbatinonusus* are most closely associated with humans. In contrast, *M. pachydermatis*, *M. nana* , *M. caprae* , *M. equina*, *M. cuniculi* , *M. brasiliensis* , *M. psittaci*, *M. vespertilionis* and *M. gallinae* have been isolated primarily from animal hosts [17, 18, 19, 20, 21, 22, 23, 24, 25].

In the present study, we describe the *Malassezia* species associated with human diseases in Brazil, identified mainly through molecular techniques. This study aims to characterise *Malassezia* spp. isolated from patients with pityriasis versicolor and seborrheic dermatitis and to evaluate their susceptibility profiles to antifungal agents.

## 2 Methodology

This study included 102 samples, comprising 78 clinically diagnosed cases of pityriasis versicolor (PV), 23 cases of seborrheic dermatitis (SD) and one sample obtained from the ear of a healthy individual. Patients were attended at the Instituto Superior de Medicina (ISMD) in the Brazilian states of Minas Gerais (59 samples) and São Paulo (31 samples). In addition, 10 samples were obtained in Tocantins, Brazil. These samples were collected between 2010 and 2013.

The study was approved by the Ethics Committees of the Universidade Federal de Minas Gerais (UFMG) and the Universidade Federal de Tocantins (UFT) (UFMG: CAAE‐0648.0.203.000‐11; UFT: CEP 139/2013), and all participants provided written informed consent.

### 2.1 Samples

Clinical samples from São Paulo were transported to Belo Horizonte in sterile packaging and subsequently cultured and isolated at the Federal University of Minas Gerais (UFMG). Samples from the state of Tocantins were initially cultured and isolated at the Federal University of Tocantins (UFT). Following isolation, these cultures were transported to UFMG in tubes containing modified Dixon's medium for further analysis. All *Malassezia* isolates included in this study are listed in Table 1 and were obtained using the same isolation procedures. All samples were processed using the same methodology described below.

**TABLE 1:** Identification of *Malassezia* species according to clinical source and location in Brazil.

Sample code (GenBank accession)	Clinical source	Antifungal treatment	Location	Species
MG215A	PV	Yes	Belo Horizonte‐MG	*M. furfur*
MG16	SD	No	Belo Horizonte‐MG	*M. furfur*
MG38	PV	NI	Belo Horizonte‐MG	*M. furfur*
MG39.2	PV	Yes	Belo Horizonte‐MG	*M. furfur*
TO1 (PZ043123)	PV	NI	Palmas‐TO	*M. furfur*
TO4 (PZ043122)	SD	NI	Palmas‐TO	*M. furfur*
TO5	PV	NI	Palmas‐TO	*M. furfur*
TO7	SD	NI	Palmas‐TO	*M. furfur*
TO8	PV	NI	Palmas‐TO	*M. furfur*
TO19	SD	NI	Palmas‐TO	*M. furfur*
MG SC1A	PV	NI	Belo Horizonte‐MG	*M. furfur*
MG16.2	SD	No	Belo Horizonte‐MG	*M. furfur*
MG16.1 (PZ043124)	SD	No	Belo Horizonte‐MG	*M. furfur*
SP78	PV	No	São Paulo‐SP	*M. furfur*
TO69.1	SD	No	Palmas‐TO	*M. furfur*
MG215B	PV	Yes	Belo Horizonte‐MG	*M. furfur*
SP28	PV	Ni	São Paulo‐SP	*M. sympodialis*
MG24	PV	No	Belo Horizonte‐MG	*M. sympodialis*
MG31B	PV	Yes	Belo Horizonte‐MG	*M. sympodialis*
MG8	PV	No	Belo Horizonte‐MG	*M. sympodialis*
MG SC1C (PZ043126)	PV	Ni	Belo Horizonte‐MG	*M. sympodialis*
MG SC1B	PV	Ni	Belo Horizonte‐MG	*M. sympodialis*
SP57 (PZ043125)	PV	Ni	São Paulo‐SP	*M. sympodialis*
MG18	Healthy skin	No	Belo Horizonte‐MG	*M. sympodialis*
SP88 (PZ043127)	PV	No	São Paulo‐SP	*M. sympodialis*
SP28.2 (PZ043128)	PV	Ni	São Paulo‐SP	*M. sympodialis*
MG53 (PZ043129)	PV	Ni	Belo Horizonte‐MG	*M. sympodialis*
MG MZ2B	PV	Ni	Belo Horizonte‐MG	*M. globosa*
MG13	SD	No	Belo Horizonte‐MG	*M. globosa*
SP87 (PZ055600)	PV	No	São Paulo‐SP	*M. globosa*
MG31A	PV	Yes	Belo Horizonte‐MG	*M. globosa*
MG25	PV	No	Belo Horizonte‐MG	*M. globosa*
MG29.1	SD	No	Belo Horizonte‐MG	*M. globosa*
MG38.1 (MH010208)	PV	Ni	Belo Horizonte‐MG	*M. japonica*
SP89.2 (PZ043131)	SD	Yes	São Paulo‐SP	*M. japonica*
SP97.1 (MH010207)	PV	No	São Paulo‐SP	*M. japonica*
SP98.1	SD	No	São Paulo‐SP	*M. slooffiae*
MG46	PV	Ni	Belo Horizonte‐MG	*M. slooffiae*
MG5464 (PZ043130)	PV	Yes	Belo Horizonte‐MG	*M. yamatoensis*
MG MZ2A (MN108127)	PV	Ni	Belo Horizonte‐MG	*M. yamatoensis*

Abbreviations: MG, Minas Gerais; Ni, Not informed; PV, pityriasis versicolor; SD, seborrheic dermatitis; SP, São Paulo.

First, a thorough clinical examination was performed to assess the characteristics and distribution of lesions, the colour and texture of the patient's skin, and the presence of any associated dermatological or systemic conditions. Subsequently, samples were collected by gently scraping the affected area with a sterile blade and examined microscopically after treatment with a 30% potassium hydroxide (KOH) solution and Parker blue ink. The samples were then inoculated onto modified Dixon's medium and incubated at 32°C for up to 30 days [26].

### 2.2 Molecular Analysis

#### 2.2.1 DNA Extraction

Genomic DNA was extracted from pure cultures. Approximately 0.2 g of cells were resuspended in lysis buffer (25 mL Tris 1 M + HCl; 5 mL EDTA 0.5 M; 10 mL NaCl 5 M; 50 mL SDS 10%; 410 mL distilled water). The samples were placed on an orbital shaker (Biosan, USA) at 250 rpm for 30 min and then incubated at 65°C for 60 min. Subsequently, 500 μL of phenol:chloroform:isoamyl alcohol (25:24:1) was added to each tube, followed by centrifugation at 9169 × g for 15 min.

The upper phase was transferred to a new microcentrifuge tube, and 65 μL of sodium acetate (3 M) and 75 μL of NaCl (1 M) were added. The mixture was incubated on ice for 30 min and centrifuged again at 9169 × g for 10 min at 4°C. The supernatant was transferred to a new tube, and 250 μL of isopropyl alcohol was added to precipitate the DNA. The precipitated DNA was centrifuged at 11,180 × g for 10 min. The supernatant was discarded, and the pellet was washed with 70% ethanol. Finally, the DNA was air‐dried and resuspended in 50 μL of TE buffer (2 mL Tris 1 M + HCl; 0.4 mL EDTA 0.5 M; 197.6 mL distilled water).

#### 2.2.2 *Malassezia* Identification

Yeast isolates were grouped and subjected to PCR fingerprinting using the microsatellite primer (GTG)_5_ [27]. Isolates showing identical DNA banding patterns were considered to belong to the same species [28]. Representative isolates from each molecular group were selected for sequencing. The D1/D2 variable domains of the large subunit (LSU) rDNA were amplified by PCR using primers NL1 and NL4, as described by Lachance et al. [29].

Sequencing was performed on an ABI 3130 capillary electrophoresis system (Applied Biosystems, USA) using BigDye v3.1 chemistry and POP7 polymer. The obtained sequences were compared with those available in the GenBank database (National Center for Biotechnology Information, NCBI) using the Basic Local Alignment Search Tool (BLAST; http://www.ncbi.nlm.nih.gov) [30].

### 2.3 Antifungal Susceptibility Testing

Yeasts were grown on modified Dixon agar for 72 h at 32°C, and inoculum suspensions were prepared as described by Rincón et al. [31]. Inocula were adjusted to 10^6^ CFU/mL and standardised spectrophotometrically to an absorbance of 1.0 at 660 nm ( *M. globosa* and *M. restricta*) or 0.425–0.435 at 530 nm (other species).

Broth microdilution testing was performed according to the Clinical and Laboratory Standards Institute (CLSI) document M27‐A3 [32], with modifications proposed by Rincón et al. [31]. Susceptibility testing was carried out in sterile flat‐bottom 96‐well microplates (Difco Laboratories, Detroit, MI, USA) using Christensen's urea broth supplemented with 0.1% Tween 80 and 0.5% Tween 40 (Sigma, USA).

Stock solutions of itraconazole and ketoconazole (Sigma‐Aldrich, USA), clotrimazole (Bayer, Germany), isoconazole and miconazole (Cristália, Brazil) were prepared in dimethyl sulfoxide, while caspofungin (CANCIDAS, Merck Sharp & Dohme, Brazil) was prepared in distilled water.

Final drug concentrations in Christensen's urea broth ranged from 0.03 to 16 μg/mL, except for clotrimazole, which ranged from 0.062 to 64 μg/mL. Microdilution plates were incubated at 32°C for 96 h ( *M. globosa* and *M. restricta*) or 72 h (other species) following Rincón et al. [31]. Endpoints were determined visually by comparing each well with the drug‐free growth control. The minimum inhibitory concentration (MIC) was defined as the lowest antifungal concentration (μg/mL) that inhibited at least 50% of growth. All assays were performed in triplicate using biological replicates. MIC values were compared among species using the Kruskal–Wallis test in GraphPad Prism (GraphPad Software, San Diego, CA, USA).

## 3 Results

A total of 40 *Malassezia* isolates were obtained from the 102 clinical samples analysed. Table 1 shows the clinical samples that yielded positive cultures. Among the 40 isolates obtained, six *Malassezia* species were identified: *M. furfur*, *M. sympodialis*, *M. globosa* , *M. japonica* , *M. slooffiae* and *M. yamatoensis* (Table 1). *M. furfur* was the most prevalent species overall (16 isolates, 40.0%), followed by *M. sympodialis* (11 isolates, 27.5%), *M. globosa* (6 isolates, 15.0%), *M. japonica* (3 isolates, 7.5%) and both *M. yamatoensis* and *M. slooffiae* (2 isolates each, 5.0%).

Species distribution varied by location. In Palmas, Tocantins, all seven isolates were *M. furfur*. In Belo Horizonte, *M. furfur* was also the most frequent (8/16 isolates), followed by *M. sympodialis* (7), *M. globosa* (5), *M. yamatoensis* (2) and both *M. slooffiae* and *M. japonica* (1 each). In São Paulo, *M. sympodialis* predominated (4/11 isolates), followed by *M. japonica* (2), with *M. furfur*, *M. slooffiae* and *M. globosa* represented by one isolate each. Regarding disease association, *M. japonica* and *M. yamatoensis* were found exclusively in patients with pityriasis versicolor, whereas the other species occurred in both pityriasis versicolor and seborrheic dermatitis.

Interestingly, mixed‐species colonisation was observed in some patients. In one patient with pityriasis versicolor, both *M. yamatoensis* (MG MZ2A) and *M. globosa* (MG MZ2B) were isolated. Co‐isolation of *M. sympodialis* (MG 31B) and *M. globosa* (MG 31A) was also observed from a single lesion in another patient. In a different patient, two distinct isolates of *M. furfur* (MG215A and MG215B) were recovered from the same lesion. Similarly, *M. sympodialis* isolates MGSC1B and MGSC1C, together with *M. furfur* (MGSC1A), were recovered from a single lesion. Notably, isolates belonging to the same species differed in colony morphology and in the molecular profiles obtained using the (GTG)_5_ primer.

The D1/D2 sequences of at least one isolate from each species were deposited in GenBank, and the corresponding accession numbers are provided in Table 1. The strains are also preserved in the Collection of Microorganisms, DNA, and Cells of the Federal University of Minas Gerais (Coleção de Microrganismos, DNA e Células da UFMG), Belo Horizonte, Minas Gerais, Brazil, where they are maintained in a metabolically inactive state.

The antifungal susceptibility profile revealed that all tested isolates exhibited high MICs to caspofungin, with values > 16 μg/mL. Isoconazole also showed elevated MICs for most isolates, with an MIC₅₀ of 8 μg/mL. Miconazole and clotrimazole presented an MIC₅₀ of 4 μg/mL, while itraconazole and ketoconazole demonstrated better activity against the tested isolates, with an MIC₅₀ of 0.125 μg/mL. Differences in MIC distributions among species were evaluated using the Kruskal–Wallis test, and no statistically significant differences were observed for any antifungal tested (*p* > 0.05). However, it should be considered that the number of isolates differs among the species, which may affect the statistical analysis.

Interestingly, several isolates displayed low susceptibility to nearly all antifungals tested, including MG215A (*M. furfur*), MG28 (*M. sympodialis*), MG MZ2B and MG31A ( *M. globosa* ), SP89.2 ( *M. japonica* ) and MG5464 (*M. yamatoensis*) (Table 2). Notably, isolate MG31A exhibited the highest MIC values across all antifungals tested. Some of these isolates were obtained from patients with a prior history of antifungal use, including MG215A, MG215B, MG MZ2B, MG31A, MGA31B, SP89.2 and MG5464 (Table 1). For several other isolates, information on previous antifungal exposure was not available.

**TABLE 2:** Susceptibility profile of *Malassezia* spp. clinical isolates to antifungal drugs using Minimal Inhibitory Concentration (MIC).

Sample code	Species	CET	ITZ	CLO	MI	ISCZ	CASP
	MIC (μg/ml)						
MG215A	*M. furfur*	16	8	16	16	16	> 16
MG16	*M. furfur*	0.125	0.125	2	0.5	4	> 16
MG38	*M. furfur*	0.0062	0.125	2	0.5	4	> 16
MG39.2	*M. furfur*	2	0.0062	1	1	4	> 16
TO1	*M. furfur*	0.031	0.25	1	0.5	0.5	> 16
TO4	*M. furfur*	0.5	0.25	32	8	16	> 16
TO5	*M. furfur*	0.25	0.125	32	8	8	> 16
TO7	*M. furfur*	0.125	0.125	8	16	16	> 16
TO8b	*M. furfur*	0.062	0.031	1	0.5	0.25	> 16
TO19	*M. furfur*	2	0.062	2	0.5	0.5	> 16
MG SC1A	*M. furfur*	2	8	2	2	1	> 16
MG16.2	*M. furfur*	0.25	0.125	8	0.5	0.125	> 16
MG16.1	*M. furfur*	0.5	0.5	1	1	0.25	> 16
SP78	*M. furfur*	1	0.125	2	8	16	> 16
MG69	*M. furfur*	1	0.125	2	8	16	> 16
MG215B	*M. furfur*	1	0.125	2	8	16	> 16
SP28	*M. sympodialis*	> 16	16	4	16	> 16	> 16
MG24.3	*M. sympodialis*	—	—	—	—	—	—
MG31B	*M. sympodialis*	—	—	—	—	—	—
MG8	*M. sympodialis*	0.031	0.062	> 64	1	0.5	> 16
MG SC1B	*M. sympodialis*	0.031	0.031	0.5	0.125	16	> 16
MG SC1C	*M. sympodialis*	0.062	0.062	4	4	4	> 16
SP57	*M. sympodialis*	4	0.062	32	0.125	16	> 16
MG18.1	*M. sympodialis*	8	0.25	0.5	1	1	> 16
SP88	*M. sympodialis*	0.125	1	16	1	8	> 16
SP28.2	*M. sympodialis*	2	0.062	4	2	8	> 16
MG53	*M. sympodialis*	0.125	4	8	4	8	> 16
MG MZ2B	*M. globosa*	> 16	0.062	4	> 16	16	> 16
MG13	*M. globosa*	0.062	0.062	0.5	0.031	0.5	> 16
SP87	*M. globosa*	1	0.125	32	2	4	> 16
MG31A	*M. globosa*	> 16	> 16	> 64	> 16	> 16	> 16
MG25	*M. globosa*	—	—	—	—	—	—
MG29.1	*M. globosa*	—	—	—	—	—	—
MG38.3	*M. japonica*	4	0.25	1	4	1	> 16
SP89.2	*M. japonica*	16	0.125	16	8	16	> 16
SP97	*M. japonica*	2	2	2	8	16	> 16
SP98	*M. slooffiae*	4	8	4	8	16	> 16
MG46	*M. slooffiae*	1	0.125	32	2	4	> 16
MG 5464	*M. yamatoensis*	16	16	4	> 16	16	> 16
MG MZ2A	*M. yamatoensis*	0.5	0.125	16	4	8	> 16
MIC 50		0.125	0.125	4	4	8	> 16

*Note:* Not tested; No statistically significant differences in MIC values were observed among the *Malassezia* species for any of the antifungal agents tested (Kruskal–Wallis test, *p* > 0.05), *p*‐values: CET = 0.287; ITZ = 0.726; CLO = 0.605; MI = 0.452; ISCZ = 0.818.

Abbreviations: CASP, Caspofungin; CET, Cetoconazole; CLO, Clotrimazole; ITZ, Itraconazole; ISCZ, Izoconazole; MI, Miconazole.

## 4 Discussion

In the present study, *M. furfur* was the most frequently isolated species from the patients analysed. Interestingly, in a study by Soares et al. [33], which investigated the *Malassezia* microbiota of healthy individuals and patients with seborrheic dermatitis in Brazil using genetic diversity analysis, *M. furfur* was not detected. Similarly, studies in Chinese seborrheic dermatitis patients reported *M. furfur* as the most frequently isolated species, corroborating our findings [34].

In contrast, Pedrosa et al. [35] reported *M. sympodialis* (*n* = 69) as the most frequent species among 86 isolates obtained from patients with pityriasis versicolor, seborrheic dermatitis and healthy volunteers at a university hospital in Porto, Portugal. In our study, *M. sympodialis* was the second most prevalent species (*n* = 11) in patients with pityriasis versicolor and seborrheic dermatitis. As highlighted by Miranda et al. [36], variations in *Malassezia* species occur across different geographical regions. We hypothesise that similar variation may exist between Brazilian states, likely influenced by regional temperature differences. The average annual temperature varies among Brazilian cities, with mean annual values of 19.3°C in São Paulo, 21.4°C in Belo Horizonte and 26.7°C in Palmas, and corresponding mean annual maximum temperatures of 25.7°C, 27.3°C and 32.3°C, respectively, according to the climatological normals (1991–2020) reported by the Brazilian National Institute of Meteorology (INMET) [37]. Some species, such as *M. furfur*, grow better at 37°C, while others, such as *M. yamatoensis*, grow optimally at 32°C [1]. In our study, *M. furfur* was predominantly isolated in the hotter cities of Belo Horizonte (MG) and Palmas (TO), whereas *M. sympodialis* was more frequently isolated in São Paulo, which has milder temperatures. Nevertheless, further studies with larger sample sizes are required to confirm these observations.

Interestingly, two uncommon species, *M. japonica* and *M. yamatoensis*, were isolated from patients with pityriasis versicolor. *M. japonica* was isolated from two patients in São Paulo and *M. yamatoensis* from two patients in Belo Horizonte. To our knowledge, this is the first report of *M. yamatoensis* isolation outside Japan [17]. According to Hiruma et al. [23], the new species identified by these authors, *M*. *polysorbatinonusus*, as causing seborrheic dermatitis in a patient in Japan is molecularly related to *M. yamatoensis*. However, this new species is unable to metabolise the tested tweens but can grow at 40°C. *M. yamatoensis*, in the present study, was also able to metabolise all the tested tweens as well as Cremophor (see Table S1). *M. japonica* has previously been reported in Japan, China, Portugal and India [18, 34, 35, 38]. Interestingly, *M. japonica* has also been isolated from the cerumen of carcasses of several non‐human primate species in Japan [39].

Although most *Malassezia*‐associated diseases can be cured without major complications, relapses have become increasingly frequent. Reports in the literature also describe treatment failures with prophylactic fluconazole and posaconazole in patients with fungemia [40]. In the present study, all tested isolates exhibited high MICs to caspofungin. Isoconazole, miconazole and clotrimazole showed moderately high MICs. Pedrosa et al. [35] also reported high MICs for clotrimazole in several *Malassezia* isolates, although they did not test isoconazole or miconazole. In contrast, itraconazole and ketoconazole showed better activity against the tested isolates in our study.

Nonetheless, some isolates displayed low susceptibility to nearly all antifungals tested, including MG215A (*M. furfur*), SP28 (*M. sympodialis*), MG MZ2B and MG31A ( *M. globosa* ), SP89.2 ( *M. japonica* ) and MG5464 (*M. yamatoensis*) (Table 2). Isolate MG31A was particularly notable, exhibiting high MICs to all antifungals tested. Moreover, isolates from patients with a history of prior antifungal use, such as MG215A, MG MZ2B, MG31A, SP89.2 and MG5464, tended to show increased resistance. Interestingly, we also observed cases in which two isolates of the same species, obtained from a single patient, displayed markedly different susceptibility profiles, as exemplified by MG215B and MG215A. In addition, isolates MGMZ2 and MGMZ4, obtained from the same clinical sample, were identified as two distinct species, *M. yamatoensis* and *M. globosa* , respectively, highlighting the coexistence of different *Malassezia* species within a single patient sample. Unfortunately, information on prior antifungal use was not available for some patients, including those from whom isolates MGMZ2B and MG28 were obtained, which also exhibited high MIC values.

## 5 Conclusion

In the present study, we observed a diverse range of *Malassezia* species causing pityriasis versicolor and seborrheic dermatitis at the studied sites in Brazil. Notably, rare species such as *M. yamatoensis* and *M. japonica* were identified among the isolates. The tested isolates generally exhibited lower MICs to itraconazole and ketoconazole; however, some isolates displayed high MICs to all antifungals evaluated. These findings underscore the importance of further studies and careful consideration in the use of antifungal agents for the treatment of diseases caused by *Malassezia* species.

## Author Contributions

**Ana Paula Possa:** investigation, data curation. **Susana Johann:** conceptualization, investigation, writing – original draft, writing – review and editing, visualization, software, formal analysis, project administration, supervision. **Diogo Coelho de Pádua Oliveira:** data curation, investigation, methodology, validation. **Raquel Vilela:** funding acquisition, writing – review and editing, data curation, formal analysis. **Ana Kleiber P. Borges:** data curation, writing – review and editing. **Patrícia Silva Cisalpino:** conceptualization, writing – review and editing, resources, formal analysis.

## Funding

This work is part of the project “INCT Yeasts: Biodiversity, preservation and biotechnological innovation”, funded by Conselho Nacional de Desenvolvimento Científico e Tecnológico (CNPq), Brazil, grant #406564/2022‐1. Funding was also provided by Conselho Nacional de Desenvolvimento Científico e Tecnológico (CNPq) (314952/2021‐7), Fundação de Amparo Pesquisa Estado de Minas Gerais (FAPEMIG) (APQ‐02944‐18), Coordenação de Aperfeiçoamento de Pessoal de Nível Superior (CAPES) and Pró‐Reitoria de Pesquisa da UFMG.

## Supporting information


**Table S1:** Utilisation of Tweens and Cremophor by *Malassezia* isolates.

## Data Availability

The data that support the findings of this study are available on request from the corresponding author. The data are not publicly available due to privacy or ethical restrictions.
