# Supplementary material for: Diversity and Antifungal Susceptibility of Malassezia spp. Isolated From Brazilian Patients With Pityriasis Versicolor and Seborrheic Dermatitis
Source: Mycoses. 2026 Mar 27;69(4):e70171. doi: 10.1111/myc.70171 (PMC13032048; doi:10.1111/myc.70171)
Supplement: Supplementary file 1 — Table S1: Utilisation of Tweens and Cremophor by Malassezia isolates. [file MYC-69-e70171-s001.docx]

| *Malassezia* | Tween 20 | Tween 40 | Tween 60 | Tween 80 | Cremophor |
| --- | --- | --- | --- | --- | --- |
| *M. yamatoensis*  5464A.1 | + | + | + | + | +*** |
| *M. yamatoensis* MZ2 | +** | + | + | + | +*** |
| *M. japonica*  A38C1 | +** | +*** | + | + | - |

Table S1. Utilization of Tweens and Cremophor by *Malassezia* isolates.

+= Growth presence; -= without growth; +**= Growth begins at a distance from the well and proceeds centripetally; there may be some diffuse colonies near the well; +***= Weak growth at a distance from the well; adapted from Guého-Kellermann E and Batra R. Boekhout (2011).

Guého-Kellermann E, Batra R. Boekhout T. *Malassezia* Baillon. In: Kurtzman C, Fell JW, Boekhout T. The Yeast: A taxonomic study, Volume III, 5th ed., Amsterdam: Elsevier Science; 2011, p. 1807 - 1832.
